# Associations Between Measures of Auditory Function and Brief Assessments of Cognition

**DOI:** 10.1044/2020_AJA-20-00077

**Published:** 2020-09-25

**Authors:** Larry E. Humes

**Affiliations:** a Department of Speech and Hearing Sciences, Indiana University, Bloomington

## Abstract

**Purpose:**

The two primary purposes of this report are (a) to compare the results of three brief cognitive screens in older adults and (b) to examine associations between performance on each of the screens and auditory function measured either concurrently or 9 years earlier.

**Method:**

This was a prospective longitudinal study of 98 adults (66 women) with baseline ages ranging from 40 to 85 years. The mean interval between T1 baseline and T2 follow-up measurements was 8.8 years with a range from 7 to 11 years. Measures of hearing threshold, gap detection, and auditory temporal-order identification were completed at T1 and T2. The Mini-Mental State Examination was completed at T1 and T2, whereas the Montreal Cognitive Assessment (MoCA) and A Quick Test were completed at T2 only.

**Results:**

Higher scores and pass rates were obtained for the Mini-Mental State Examination than for the MoCA or the A Quick Test. The measures were moderately correlated among themselves and with the Wechsler Adult Intelligence Scale–Third Edition. Significant associations emerged frequently between auditory and cognitive functions, most often for the auditory measure of temporal-order identification, including dichotic measures of this ability.

**Conclusions:**

From this evaluation, the MoCA emerged as the preferred test for clinicians desiring a quick assessment of the cognitive function of their older patients. Auditory temporal-order identification is associated with cognitive function and explains about 10%–20% of the variation in cognitive function independent of age and hearing loss.

**Supplemental Material:**

https://doi.org/10.23641/asha.12986021

There has been a long-standing interest in the associations between age-related declines in auditory and cognitive functions (Danielsson et al., 2019; Humes & Young, 2016; Lindenberger & Baltes, 1994; Pronk et al., 2019; Rönnberg et al., 2011, 2014; Schneider & Pichora-Fuller, 2000; Wayne & Johnsrude, 2015). The interest in this topic over the past 2 decades was heightened when the work of F. R. Lin et al. found significant linkages between hearing loss and dementia in older adults (e.g., F. R. Lin et al., 2011, 2013). A flurry of research activity since then has led to a sufficient number of published articles to generate systematic reviews and meta-analyses. Two recently published reviews, one focusing on dementia in older adults (Livingston et al., 2017) and the other evaluating both healthy aging and cognitive disorders (Loughrey et al., 2018), concluded that preexisting hearing loss consistently emerges as one of the strongest modifiable factors, typically accounting for about 5%–10% of the variance in cognitive function.

Often, the cognitive measures required for inclusion of studies in such systematic reviews have been all or parts of full diagnostic assessments of cognitive function, such as scales of the Wechsler Adult Intelligence Scale (Wechsler, 1955, 1981, 1997, 2008). Due to the professional requirements for the administration of such tests and the length required for test completion and scoring, these tests are not practical for most clinical audiologists to administer. Yet, there is mounting evidence in support of links: (a) between hearing loss and cognitive function in older adults (Livingston et al., 2017; Loughrey et al., 2018), even when hearing thresholds are within the conventional normal-hearing range (Golub et al., 2020); (b) between cognitive function and aided speech understanding (e.g., Humes, Kidd, & Lentz, 2013); and (c) possibly between hearing aid intervention and reduction in cognitive decline (Amieva & Ouvrard, 2020). It is likely to be increasingly important for clinical audiologists to have access to one of several brief cognitive screens that have been developed with older adults in mind.

Recognizing the importance of widespread reliable and valid identification of older adults with cognitive difficulties, several brief cognitive screens have been developed. The brief cognitive test that has the longest history in application to older adults is the Mini-Mental State Examination (MMSE; Folstein et al., 1975). Two additional brief cognitive screens that have emerged more recently are the Montreal Cognitive Assessment (MoCA; Nasreddine et al., 2005) and A Quick Test (AQT; Wiig et al., 2002). Whereas both the MMSE and the MoCA afford broad assessments of various cognitive domains, the AQT focuses specifically on cognitive verbal processing speed (PS). There is also evidence of an association between hearing loss and cognitive function using the MMSE (Deal et al., 2017; Fischer et al., 2016; F. R. Lin et al., 2011, 2013) and the MoCA (Dupuis et al., 2015; Gosselin et al., 2019). Although the effects of aging on AQT have been documented (Wiig et al., 2007), research specifically documenting the effects of hearing loss on AQT scores could not be found.

All three of these brief cognitive screens were completed on a group of 98 middle-aged and older adults. This same group of 98 adults had completed a variety of psychoacoustic tests and full cognitive assessments (Wechsler Adult Intelligence Scale–Third Edition [WAIS-III]) both 9 years earlier and at the same time as completion of the brief cognitive screens. Of the brief cognitive screens, only one (MMSE) was administered both at the T1 baseline and again 9 years later at the T2 follow-up. The original cross-sectional study (Humes et al., 2013) included 203 adults who were between 40 and 87 years old at the T1 baseline. This report provides the results for 98 adults, 48.3% of the original cohort, who returned for the 8- to 9-year longitudinal follow-up study. The specific auditory measures completed at T1 and T2 included (a) clinical measures of hearing threshold from 250 to 8000 Hz bilaterally; (b) psychophysical measures of hearing threshold at 500, 1400, and 4000 Hz; (c) psychophysical measures of gap-detection (GapDet) threshold for 1000-Hz bands of noise centered at 1000 and 3500 Hz; and (d) four measures of temporal-order (TempOrd) identification of brief vowel sequences presented either monaurally or dichotically. In addition to these auditory measures, the full WAIS-III (W3; Wechsler, 1997) and the MMSE were completed at T1 and T2. The focus here is on the three brief cognitive assessments, and the W3 measures are used only to evaluate the validity of the brief cognitive screens. The questions addressed below are as follows:

At T2, what are the associations among the brief cognitive screens and how is each related to the results of the more detailed cognitive assessment provided by the W3?

Using established pass–fail criteria for each cognitive screen, do the same participants pass or fail on all three screens at T2?

Is auditory function, either 9 years prior at baseline (T1) or at follow-up (T2), associated with T2 cognitive function as measured with these brief tools?

Are the *rates* of decline in auditory function or baseline T1 auditory measures predictive of the rate of decline in MMSE scores over the 9-year period?

These questions will be addressed primarily through a series of correlational and linear multiple-regression analyses examining associations among the measures.

## Method

### Participants

A total of 98 adults (66 women, 32 men), with a mean age of 62.6 years at baseline, participated in this longitudinal follow-up study. Participants ranged in age from 47 to 94 years, with a mean age of 71.5 years, at follow-up. The mean interval between T1 baseline and T2 follow-up measurements was 8.8 years with a range of 7–11 years. Most (76.5%) were retested 9 years following baseline, an additional 14.3% within 1 year of the 9-year retest interval. Given these variations in test–retest or T1–T2 interval, this will often be treated as a covariate in the analyses of rates of decline over the T1–T2 time period.

On most baseline (T1) measures included in the longitudinal follow-up study, there were no significant differences (*p* > .05; independent-samples *t* tests, uncorrected for multiple comparisons) between the 98 returnees and the 105 nonreturnees. The two groups did not differ significantly (*p* > .05) in age at baseline evaluation, with the returnees having a mean age at baseline of 62.6 years and the nonreturnees 65.3 years. The two groups also did not differ significantly (*p* > .05) regarding baseline scores on the MMSE. The two groups *did* differ significantly (chi-square test, *p* < .05) regarding the proportion of men and women: 54 of 105 (51%) nonreturnees and 66 of 98 (67%) of the returnees were women. Regarding baseline (T1) differences among the auditory and cognitive measures described in more detail below, nonreturnees had significantly worse baselines for auditory GapDet threshold at 1000 Hz, raw block-design W3 subscale scores, and raw matrix-reasoning W3 subscale scores. All told, these differences amount to significant differences (*p* < .05) in T1 baseline performance between returnees and nonreturnees on only one of 18 pure-tone audiometric measures, one of nine auditory psychophysical measures, and two of 13 W3 subscale scores. It is noteworthy, though, for those few measures showing significant group differences in baseline performance, it was always with the 98 returnees outperforming the 105 nonreturnees. Nonetheless, the two groups were much more similar than not. We conclude that the 98 returnees are a representative sample of the original baseline cohort of 203 adults.

Participants were recruited from the local community, rather than from the university clinic, and had a wide range of hearing levels. The mean audiograms at T1 and T2 revealed bilateral sloping mild-to-moderate hearing loss with about an 8- to 10-dB progression of hearing loss from T1 to T2. The mean bilateral high-frequency pure-tone average, the mean of thresholds at 1000, 2000, and 4000 Hz in both ears, was 23.3 dB HL at T1 and 31.2 dB HL at T2, which represents a significant decline in hearing, *t*(97) = 13.0, *p* < .001. Of the 98 participants, 19 were wearing hearing aids at T2, six of whom were also wearing hearing aids at T1.

Informed consent was obtained from all 98 participants, and they were paid $12/hr for their participation. This study was approved by the Indiana University Bloomington Institutional Review Board.

### Materials and Procedures

During the initial session of the baseline and follow-up studies, audiological examinations were completed, along with the MMSE. The subjects next completed the full W3 yielding the 13 standard scale scores and, in a later session, the MoCA and the AQT. Raw W3 scores, rather than age-corrected scores, are used throughout this report. All cognitive testing was completed by a trained examiner in a face-to-face session. All hearing aid wearers wore their hearing aids during the cognitive testing. In addition, an assistive device (a hard-wired microphone, amplifier, and headset assembly) was available for anyone expressing difficulty hearing the examiner, but this was never used or requested.

Next, the measures of auditory threshold sensitivity and GapDet were completed. For auditory threshold measurement, measures were obtained first at 500 Hz, then at 1400 Hz, and finally at 4000 Hz. Similarly, measurement of GapDet threshold began at the 1000-Hz center frequency and then proceeded to the 3500-Hz center frequency. This use of a fixed order reinforced the need for familiarization trials prior to each measure and for stable threshold estimates based on 200–250 trials. Next, TempOrd identification measures were completed. Four TempOrd identification tasks were completed. Three of the four tasks required the identification of two-item sequences (out of the four possible stimuli), and one required the identification of a four-item sequence. The 3 two-item sequences differed regarding how the stimuli were presented to the subject with stimuli in the sequence presented either to the same ear (monaural) or to different ears (dichotic). This manipulation was designed to explore lower level (peripheral) versus higher level (central) auditory temporal-processing mechanisms. For example, for the auditory two-item dichotic task, the two sensory inputs cannot interact until the first auditory center in the brainstem processes inputs from both ears (the superior olivary complex). On the other hand, the same-ear monaural version of this task makes it possible for interaction of the two stimuli in the sequence at a much lower level, as low as the cochlea. For the two dichotic, two-item tasks, the difference between them was in the response required of the subject. In one case, the subject was required to identify the stimulus sequence, just as in the monaural version of this task, whereas in the other case, the task was simply to identify which ear (right or left) was stimulated first. Finally, the four-item sequence was included to increase the cognitive demands for this TempOrd identification task, thereby increasing the likelihood for uncovering a common underlying cognitive factor (Fogerty et al., 2016). For all these auditory TempOrd measures, the threshold estimate obtained was the stimulus onset asynchrony (SOA) that was approximately midway between chance and 100% correct performance on the psychometric function relating performance to SOA. Further details regarding the stimuli and procedures can be found elsewhere (Fogerty et al., 2010; Humes et al., 2010).

#### Auditory Procedures and Equipment

All auditory psychophysical testing was completed in a sound-attenuating booth meeting the American National Standards Institute (ANSI) S3.1 standard for “ears covered” threshold measurements (ANSI, 2003). Two adjacent subject stations were housed within the booth. Each participant was seated comfortably in front of a touch-screen display (Elo Model 1915L). The right ear was the test ear for all monaural measurements in this study. Stimuli were generated off-line and presented to each listener using the custom MATLAB software. Stimuli were presented from the Tucker–Davis Technologies (TDT) digital array processor with 16-bit resolution at a sampling frequency of 48828 Hz. The output of the digital-to-analog converter was routed to a TDT programmable attenuator (PA-5), to a TDT headphone buffer (HB-7), and then to an Etymotic Research 3A insert earphone. Each insert earphone was calibrated acoustically in an HA-1 2-cm^3^ coupler (Frank & Richards, 1991). Output levels were checked electrically just prior to the insert earphones at the beginning of each data collection session and were verified acoustically using a Larson Davis Model 2800 sound-level meter with linear weighting in the coupler monthly throughout the study. Prior to actual data collection in each experiment, all listeners received 10–30 practice trials to become familiar with the task. These trials could be repeated a second time to ensure comprehension of the tasks, if desired by the listener, but this was seldom requested. All responses were made on the touch screen and were self-paced. Correct/incorrect feedback was presented after each response during experimental testing. Further methodological details, specific to each measure, follow.

*Hearing thresholds and GapDet thresholds.* Auditory thresholds were measured for three pure-tone frequencies, namely, 500, 1414, and 4000 Hz. Stimuli were 500 ms in duration from onset to offset and had 25-ms linear rise–fall times. The maximum output for the pure-tone stimuli was 98, 100, and 101 dB SPL at 500, 1414, and 4000 Hz, respectively. Further attenuation was provided via the programmable attenuator under software control during the measurement of auditory thresholds. Two auditory GapDet measurements were made, each with a different 1000-Hz wide band of noise. These noise bands served as the stimuli with one band centered arithmetically at 1000 Hz (500–1500 Hz) and the other centered at 3500 Hz (3000–4000 Hz). Each noise band had a duration from onset to offset of 400 ms with 10-ms linear rise–fall times. A catalogue of 16 different noise bands was generated for each frequency region. When a temporal gap was present in a noise band, it was centered at 300-ms post stimulus onset. This temporal location of the gap is more sensitive to age effects than a location centered in the noise stimulus (Harris et al., 2010). Gap durations varied from 2 to 40 ms in steps of 2 ms and were generated by zeroing the waveform at that temporal location, which necessitated the use of a background noise that covered a broad spectrum. This ensured that the cue available to the listener for GapDet was temporal and not spectral in nature. The spectrum level of the background noise was adjusted to be 12–15 dB below that of the stimulus noise bands. The background noise began slightly before the first interval and ended slightly after the last interval for a total duration of 2.4 s. An overall presentation level of 91 dB SPL was used for each noise band and for all listeners in this study. A relatively high presentation level was used given the likelihood of significant threshold elevations in many of the older adults, especially at the higher frequencies. Additional details of stimulus construction and calibration can be found in Humes et al. (2009).

Threshold measurements were completed prior to GapDet measurements for all listeners. For measures of threshold sensitivity, an adaptive two-interval, two-alternative forced-choice paradigm was employed. Listeners simply selected the interval (marked by a rectangular box on a visual display) that contained the signal with an a priori probability of .5 that the signal would be in either Interval 1 or Interval 2. Signal amplitude was varied adaptively from trial to trial to bracket the 70.7% and 79.3% correct points on the psychometric function using two interleaved tracks (Levitt, 1971). Three estimates each of 70.7% and 79.3% correct performance were obtained for a given signal frequency. These six performance estimates were averaged to provide a single threshold estimate corresponding to approximately 75% correct on the psychometric function. For measures of GapDet thresholds, gap duration was varied using the same interleaved adaptive tracking procedures as those described for the threshold measurements, including performance levels tracked (70.7% and 79.3%). In addition, for these measurements, a three-interval, two-alternative forced-choice paradigm was used as described more fully in Humes et al. (2009). The stimulus waveforms in a given trial were identical except that a temporal gap had been inserted into the stimulus presented during comparison Intervals 1 or 2. The specific noise-band waveform used on a given trial, however, was randomly selected among the 16 available in a stimulus catalogue. The listener's task on each trial was to select the comparison interval that contained the gap or that differed from the standard (which never contained a gap).

*Auditory TempOrd measures.* For the four auditory TempOrd identification tasks, four confusable vowel stimuli /I, e, a, u/ were recorded by a male talker in a sound-attenuating booth using an Audio-Technica AT2035 microphone. Vowels were produced in a /p/-vowel-/t/context. Productions of four vowels that had the shortest duration, F2 < 1800 Hz, and good identification during piloting were selected for stimuli. Stimuli were digitally edited to remove voiceless sounds, leaving only the voiced pitch pulses, and modified in MATLAB using Speech Transformation and Representation by Adaptive Interpolation of weiGHTed spectrogram (STRAIGHT) (Kawahara et al., 1999) to be 70 ms long with a fundamental frequency of 100 Hz. Stimuli were low-pass filtered at 1800 Hz and normalized to the same root-mean-square level. Low-pass filtering was used to minimize the influence of possible high-frequency hearing loss of the older adults on their vowel-identification performance. The system was calibrated using a calibration vowel of the same root-mean-square amplitude as the test stimuli, but with a duration of 3 s. A single stimulus presentation measured 83 (± 2) dB SPL, and a presentation of two overlapping stimuli measured 86 (± 2) dB SPL. All participants were required to pass an identification screening of the four vowel stimuli in isolation with at least 90% accuracy on one of up to four 20-trial blocks.

All listeners completed the four TempOrd tasks in the following order: monaural two-item identification, monaural four-item identification (Mono4), dichotic two-item vowel identification, and dichotic two-item ear or location identification (DichLOC). For all four tasks, the same vowel was never repeated twice in a row. The Mono4 task had the additional stipulation that each sequence must contain at least three of the four vowel stimuli. For the three vowel-identification tasks, listeners were required to identify, using a closed-set button response, the correct vowel sequence exactly (i.e., each vowel in the order presented) for the response to be judged correct. The ear-identification task, DichLOC, only required the listener to identify which ear (“right” or “left”) was stimulated first. The dependent variable measured was the SOA between the presented vowels. The minimum SOA values were required to begin at or above 2 ms to ensure a sequential presentation for the stimuli. For the four-item sequences, the SOA defined the onset asynchrony between successive stimulus pairs in the sequence. For example, an SOA of 10 ms indicates that the onset of the second vowel followed the onset of the first vowel by 10 ms, the onset of the third vowel followed the second vowel by 10 ms, and the onset of the fourth vowel followed the onset of the third vowel by 10 ms. A schematic of the stimulus sequences for all four TempOrd identification tasks, together with audio examples for the two monaural tasks, is provided in Supplemental Material S1.

All TempOrd tasks used the method of constant stimuli to measure the psychometric function relating percent correct identification performance to SOA. Threshold was defined as 50% correct (75% correct for DichLOC given two possible responses). Experimental testing was conducted in two stages because of large variability between listeners. The first stage consisted of a preliminary wide-range estimate of SOA threshold (i.e., using a large step size, 25 ms), whereas the second stage consisted of narrow-range testing centered at an individual's estimated wide-range threshold (i.e., using a smaller step size, 10 or 15 ms) to provide the actual SOA threshold estimates reported in the results. In the end, each threshold estimate for each TempOrd task was based on three valid narrow-range estimates that were averaged together for analysis, resulting in a total of 216 (monaural two-item identification), 288 (Mono4), or 432 (dichotic two-item vowel identification, DichLOC) trials per SOA threshold estimate.

### Data Analyses

Prior to data analyses, the results were examined for outliers for the nine psychophysical and the 13 W3 cognitive measures. SPSS (Version 26) was used to identify major outliers. Major outliers were defined as falling more than 3 times the interquartile range above the third quartile or below the first quartile for that measure. For example, assume a first quartile for some measure of 75 ms and a third quartile on that same measure of 100 ms, then the interquartile range would be 25 ms and values less than 0 ms or greater than 175 ms would be considered to be major outliers. Three or fewer, of 98, data points were identified and disregarded as major outliers for 20 of the 22 measures, with 0 major outliers identified for 14 of the 22. Major outliers appeared to be random with different participants exhibiting these extreme performance levels across measures, with outliers sometimes appearing in the original baseline measures and other times in the follow-up measures. The lone exception to this summary of outliers was the measured GapDet threshold at 1000 Hz. Here, four baseline and six follow-up measures were identified as major outliers and disregarded. Even here, however, 94 of 98 baseline and 92 of 98 follow-up 1000-Hz GapDet thresholds were retained for subsequent analyses.

## Results and Discussion

### At T2, What Are the Associations Among the Brief Cognitive Screens and How Is Each Related to the Results of the More Detailed Cognitive Assessment Provided by the W3?

To address these initial questions, the 13 W3 scale scores obtained at follow-up first were subjected to principal-component (PC) factor analysis (Gorsuch, 1983) for data reduction. This statistical procedure basically identifies clusters of correlated measures. Within each cluster of correlated measures, a common underlying factor is assumed and, based on the measures within each cluster, the researcher assigns a label to that subgroup of measures. Humes (2003) provides a tutorial on the application of this analysis approach to audiology. The main purpose of this analysis is to reduce large sets of measures to a smaller, more manageable set while not discarding important information about individual differences. The analysis is not always successful, and there are standard measures available to assess the quality of the fit. Here, a good fit was obtained as evidenced by the Kaiser-Meyer-Olkin (KMO) measure of sampling adequacy equals 0.86, all communalities ≥ 0.56, and 67.1% of the variance explained by three orthogonal (varimax rotation) components. The three factors were easily identified as a Processing Speed/Perceptual Organization (PSPO) factor, a Verbal Comprehension (VC) factor, and a Working Memory (WM) factor. The 13 W3 scale scores have now effectively been reduced to three factor scores facilitating subsequent analyses.

Table 1 provides the minimum and maximum scores observed, the means, and the standard deviations for the MMSE, MoCA (scores adjusted for education level), and AQT at the T2 follow-up. Of these brief cognitive measures, only the MMSE was included in the baseline (T1) measures and served as an exclusion criterion (if scores were less than 26).

**Table 1.** Brief cognitive measures obtained from study participants in the T2 follow-up visit.

Brief cognitive measure	*N*	Minimum	Maximum	*M*	*SD*
AQT Color & Shape time in seconds	98	38	92	57.9	12.6
MMSE score	97	23	30	28.4	1.6
MoCA score (education adjusted)	93	20	30	26.4	2.7

*Note.* AQT = A Quick Test; MMSE = Mini-Mental State Examination; MoCA = Montreal Cognitive Assessment.

Table 2 shows the correlations among the three T2 W3 PC factor scores, the three T2 brief measures of cognitive health, and age at T2 follow-up, above the diagonal. Given the significant correlations of age with several of these measures (far right column), partial correlations controlling for age were also calculated, and these appear below the diagonal in Table 2. These partial correlations reveal the associations among these cognitive measures that are *independent of age effects*. Significant correlations appear in bold font, and several patterns are noteworthy among the significant correlations. First, considering the correlations in the upper portion of the matrix in Table 2, age at follow-up is correlated with performance on all but two of the six cognitive measures, with the correlation involving W3 PSPO PC being quite strong (*r* = −.63). As noted above, this is not surprising as aging is known to impact these PS and perceptual organization cognitive measures (Salthouse, 2010a). Also, it is not surprising that the correlation between age and W3 VC PC is near zero. VC is a cognitive ability that remains intact throughout adulthood (Salthouse, 2010a). On the other hand, it is a bit surprising that age is not significantly correlated with the W3 WM PC factor scores as WM is a cognitive ability that declines throughout the adult life span (Salthouse, 2010a). The nonsignificant correlation may be due to the focus here on a limited range of the adult life span, 47–94 years old, with a T2 interquartile range of 62–80 years (e.g., Hofer et al., 2006).

**Table 2.** Correlations among the three brief clinical cognitive measures, the three WAIS-III (W3) orthogonal principal components (PCs), and age.

	W3 PC PSPO	W3 PC VC	W3 PC WM	MMSE	MoCA	AQT	T2 Age
W3 PC PSPO		.000	.000	.223*	.498**	−.506**	−.630**
W3 PC VC	−.006		.000	.425**	.318**	.092	−.008
W3 PC WM	−.099	−.001		.260*	.259*	−.289**	−.121
MMSE	.103	.435**	.240*		.466**	−.015	−.229*
MoCA	.367**	.339**	.232*	.422**		−.233*	−.370**
AQT	−.394**	.101	−.265*	.070	−.120		.347**

*Note.* The *N* was 93 for the MoCA pairs, 97 for the MMSE pairs, and otherwise 98. Pearson *r* correlations are shown above the diagonal, and partial correlations controlling for age are shown below the diagonal. Significant correlations are shown inbold font with one one (*, *p* < .05) or two (**, *p* > .01) asterisks. Wechsler Adult Intelligence Scale–Third Edition; MMSE = Mini-Mental State Examination; MoCA = Montreal Cognitive Assessment; AQT = A Quick Test; PSPO = Processing Speed/Perceptual Organization.

The upper portion of the correlation matrix in Table 2 also shows that the three brief measures of cognitive health are significantly correlated with age and in the expected directions given the nature of each test. The AQT measure is a measure of the time required to complete the task, and longer times reflect poor verbal PSs. The positive correlations with age reflect worsening performance on the AQT with advancing age. The MMSE and MoCA, on the other hand, have maximum scores of 30, with lower scores reflecting poorer performance on a variety of cognitive skills assessed with each test. The partial correlations controlling for age, shown below the diagonal in Table 2, indicate that the MoCA and MMSE are moderately correlated (*r* = .422), although not as strongly as seen in broader and larger samples (e.g., Nasreddine et al., 2005). Also, based on the partial correlations in the lower portion of the matrix in Table 2, the MoCA more broadly assesses all dimensions of the W3, whereas the MMSE does not appear to tap cognitive PS (*r* = .103, *p* > .05). The AQT, on the other hand, as expected, is mainly associated with the W3 PSPO PC (*r* = −.394) and significantly, but more weakly, associated with the W3 WM PC (*r* = −.265).

### Using Established Pass–Fail Criteria for Each Cognitive Screen, Do the Same Participants Pass or Fail on All Three Screens at T2?

The brief cognitive measures, MMSE, MoCA, and AQT, have established cut-points for “normal” or “pass” versus “abnormal” or “fail” performance. For the MMSE, scores less than 26 at T2 were considered a “fail” (Folstein et al., 1975). For the MoCA, scores less than 26 are also considered a “fail,” but a point is added to the MoCA score for those having 12 or fewer years of education (Nasreddine et al., 2005; https://www.moca.org). For the AQT, times for the third test, the color-and-shape combined test, are considered a “fail” if they exceed 70 s (Kvitting et al., 2013; Nielsen et al., 2004).

Table 3 shows the pass–fail results for each of the three paired comparisons of the cognitive screens. Within Table 3, there are three 2 × 2 comparisons of pass/fail decisions. The top two compare the MMSE pass/fail results to the MoCA (left) and AQT (right) pass/fail results. The bottom 2 × 2 matrix is a comparison of the pass/fail decisions for the MoCA to the AQT. Chi-squared analyses of each of the three 2 × 2 comparisons in Table 3 revealed that the MMSE results differed from both the MoCA, χ^2^(1) = 0.66, *p* > .10, and the AQT, χ^2^(1) = 0.26, *p* > .10, but that the AQT and MoCA results did not differ significantly, χ^2^(1) = 8.5, *p* < .01. Based on these results and the preceding correlational analyses, the MMSE might be combined with the AQT to form a more complete battery, including the assessment of cognitive PS. Given that the MoCA taps all three cognitive domains in the preceding correlation analyses (based on the partial correlations in the lower half of Table 2) and has classification accuracy that does not differ significantly from the AQT, a more efficient approach would be to just administer the MoCA. It has been demonstrated previously, moreover, that the MoCA is more sensitive to the presence of mild cognitive impairment than the MMSE (Hoops et al., 2009; Nasreddine et al., 2005; Roalf et al., 2013). In addition, a version of the MoCA for adults with severely impaired hearing has also been developed (V. Y. W. Lin et al., 2017).

**Table 3.** Numbers of participants passing and failing each of the three cognitive screens in this study.

	MoCA		AQT	
**MMSE**	Pass	Fail	Pass	Fail
Pass	56	24	69	16
Fail	7	5	9	3
**AQT**				
Pass	56	19		
Fail	7	11		

*Note.* MoCA = Montreal Cognitive Assessment; AQT = A Quick Test; MMSE = Mini-Mental State Examination.

### Is Auditory Function, Either 9 Years Prior at Baseline (T1) or at Follow-Up (T2), Associated With T2 Cognitive Function as Measured With These Brief Tools?

Next, predictors of failure for each of the brief cognitive screens were examined, considering both T1 and T2 auditory predictors, together with age, as potential predictors of failure. Prior to doing so, the auditory data from T1 and T2 were subjected to separate PC factor analyses with all audiometric thresholds (in dB HL re: ANSI, 2004) and psychophysical measures, except for the monaural four-item TempOrd SOAs (Mono4), which were eliminated due to the high percentage of missing data due to reaching the SOA limits for that measure. Again, the purpose of this preliminary factor analysis is to reduce the number of measures available to a more manageable number without losing information about individual differences in function. In this case, there were 27 auditory measures available at T1 and T2: 18 pure-tone thresholds (nine frequencies for each ear), three laboratory measures of hearing threshold, two measures of GapDet, and four measures of TempOrd identification. The data-reduction process via PC factor analysis was successful for both the T1 and T2 sets of auditory measures. For the T1 auditory analysis, a good fit was obtained with the KMO sampling adequacy equals 0.88, all communalities ≥ 0.60, and 80.8% of the variance explained by five orthogonal (varimax rotation) components: hearing loss at and above 2000 Hz bilaterally, hearing loss below 2000 Hz in the right ear, hearing loss below 2000 Hz in the left ear, GapDet, and TempOrd identification. For the factor analysis of the T2 auditory measures, a good fit was again obtained with the KMO sampling adequacy equals 0.90, all communalities ≥ 0.55, and 82.9% of the variance explained by the same five orthogonal (varimax rotation) components observed in the T1 analysis.

It is noteworthy that separate PCs emerged from this analysis of the auditory measures for hearing loss (three PCs), GapDet, and TempOrd identification. This indicates that there was no correlation of GapDet or TempOrd performance with hearing loss. Importantly, hearing loss was a separate factor that emerged rather than one common to all measures in part because great care was taken to minimize the impact of hearing loss on the other auditory measures by selecting relatively high presentation levels and minimizing spectral overlap of the stimuli with the expected region of hearing loss.

The five factor scores for T1 and T2 auditory performance were then entered into logistic regression analyses, together with *z*-transformed ages (at T1 age for T1 predictors and at T2 age for T2 predictors), with T2 cognitive pass/fail as the dependent measure. Logistic regression is ideal when the dependent measure is binary, in this case either pass or fail. No successful fits (*p* > .05) were obtained for either the T1 or T2 sets of predictors for the MMSE or the AQT. This is due, in large part, to the high percentages of “pass” for both of these tests such that classification accuracy is very high simply by designating everyone as “pass,” 87.6% and 80.6% accuracy for the MMSE and AQT, respectively. For the MoCA, however, doing so would only result in 66.7% accuracy and there is considerable room for improvement via inclusion of predictor variables. A significant logistic regression solution was obtained using both the prior T1 and the concurrent T2 predictors. For T1 predictors, χ^2^(6) = 20.6, *p* < .01, the only significant predictor was the T1 age *z* score, Wald χ^2^(1) = 5.17, *p* < .05, with the *B* coefficient = 0.83 (*SE* = 0.37). The odds ratio for T1 age was 2.3. For concurrent T2 predictors, χ^2^(6) = 26.2, *p* < .001, the only significant predictor of MoCA pass/fail was the auditory TempOrd processing factor score, Wald χ^2^(1) = 6.6, *p* < .05, with a *B* coefficient = 0.87 (*SE* = 0.34). The odds ratio for T2 TempOrd processing was 2.4. Thus, poor auditory TempOrd processing essentially doubled the odds of failing the MoCA in this group of 98 older adults.

After tallying the “fails” for each test, the total failures across all three tests were determined for 92 of the 98 participants who had completed all three brief cognitive measures. These tallies could vary from 0 to 3, and there were 50, 27, 12, and three individuals who had failed zero, one, two, or three of the brief cognitive tests, respectively. For the 27 who only failed one cognitive screen, most often, 52% of the time, it was the MoCA that was failed, with the other two measures contributing equally to screen failures. When two or more cognitive screens were failed, 80%–93% of the time, either the AQT or the MoCA was one of those tests, whereas the MMSE was one of the failed screens only 40% of the time. Thus, the MoCA appears to be most sensitive to gradations in cognitive performance at T2 among older adults, consistent with prior findings from larger scale studies (Hoops et al., 2009; Nasreddine et al., 2005; Roalf et al., 2013). The MoCA is also the brief screen that taps into the broadest range of cognitive functions (see Table 2).

### Is Current Cognitive Function at T2 Related to Auditory Function Measured 9 Years Earlier at T1?

Some models of the association between sensory and cognitive decline over the adult life span, such as the deprivation model, suggest that sensory decline precedes cognitive decline (Humes & Young, 2016; Lindenberger & Baltes, 1994; Pronk et al., 2019; Schneider & Pichora-Fuller, 2000; Uchida et al., 2019; Wayne & Johnsrude, 2015). Here, rather than examining the association between auditory function and the binary pass/fail for a given test, as was addressed above, the focus is on the association between auditory performance and *the score* obtained on each of the brief cognitive measures.

Three linear multiple-regression analyses were next performed using *z*-transformed scores for each of these brief clinical measures as the dependent measure and the five T1 auditory PC factor scores, together with *z*-transformed T1 age. The results are provided in Table 4. For the MMSE at T2, shown in the upper portion of Table 4, the lone significant predictor, *F*(6, 90) = 2.8, *p* < .05, was the T1 TempOrd factor score (T1 PC TempOrd) with the regression equation accounting for 10.0% of the variance (adjusted *r* ^2^). For the MoCA, shown in the middle of Table 4, a significant regression solution was obtained, *F*(6, 86) = 3.29, *p* < .01, accounting for 13.0% of the variance, with age as the only significant predictor. The contributions of T1 auditory TempOrd processing, however, approached significance (*p* = .056), with partial and part (semipartial) correlations only slightly lower than those for age (see Table 4). For the final brief cognitive measure, the AQT, the regression equation was significant, *F*(6, 91) = 2.77, *p* < .05, and accounted for 9.9% of the variance (adjusted *r* ^2^) but, as indicated in the lower portion of Table 4, no beta coefficients were significant. From the partial and part correlations in the right-hand columns of Table 4, it appears that weak and roughly equal contributions to the regression equation for the AQT scores were made by age, high-frequency hearing loss bilaterally, low- and middle-frequency hearing loss in the right ear, and TempOrd processing. The part and partial correlations control for the effects of the other independent variables to provide a better picture of the *independent* association of that measure with the dependent variable. In summary, various combinations of age and auditory TempOrd processing obtained 9 years prior (T1) were significant predictors of subsequent (T2) performance on these brief measures of cognitive function. When the auditory measures emerged as significant predictors, the variance explained by these factors was relatively small, typically under 10%.

**Table 4.** Results of multiple linear-regression analyses with baseline (T1) auditory principal component (PC) factor scores and *z*-transformed age as the independent variables and the dependent measures of follow-up (T2) *z*-transformed MMSE (top), MoCA (middle), and AQT (bottom) scores.

Dependent variable	Standardized	*t*	Sig.	Correlations		
	Beta			Zero-order	Partial	Part
**T2 MMSE**						
(Constant)		−0.007	.994			
T1Zage	−.165	−1.310	.193	−.226	−.137	−.127
T1PC HFHLbil	−.027	−0.237	.813	−.111	−.025	−.023
T1PC LMFHLrt	.060	0.570	.570	.009	.060	.055
T1PC LMFHLlt	.093	0.951	.344	.077	.100	.092
T1PC TempOrd	−.306	−3.014	**.003**	−.345	−.303	−.292
T1PC GapDet	.052	0.537	.592	.059	.057	.052
**T2 MoCA**						
(Constant)		−0.012	.990			
T1Zage	−.319	−2.487	**.015**	−.375	−.259	−.242
T1PC HFHLbil	−.045	−0.389	.698	−.209	−.042	−.038
T1PC LMFHLrt	.052	0.495	.622	−.049	.053	.048
T1PC LMFHLlt	−.032	−0.326	.745	−.073	−.035	−.032
T1PC TempOrd	−.197	−1.940	.056	−.271	−.205	−.189
T1PC GapDet	.061	0.627	.532	.064	.068	.061
**T2 AQT**						
(Constant)		0.000	1.000			
T1Zage	.199	1.585	.117	.343	.164	.153
T1PC HFHLbil	.134	1.164	.248	.233	.121	.112
T1PC LMFHLrt	.143	1.379	.171	.205	.143	.133
T1PC LMFHLlt	.088	0.903	.369	.107	.094	.087
T1PC TempOrd	.104	1.032	.305	.152	.108	.100
T1PC GapDet	.022	0.225	.822	.018	.024	.022

*Note.* Significant t-test p values are shown in bold font. MMSE = Mini-Mental State Examination; HFHLbil = hearing loss at and above 2000 Hz bilaterally; LMFHLrt = hearing loss below 2000 Hz in the right ear; LMFHLlt = hearing loss below 2000 Hz in the left ear; MoCA = Montreal Cognitive Assessment; AQT = A Quick Test.

### Is Current Cognitive Function at T2 Related to Auditory Function Measured at the Same Time (T2)?

To answer this question, three linear multiple-regression analyses were next performed using *z*-transformed scores for each of these brief T2 cognitive measures as the dependent measure and the five auditory PC factor scores for auditory function from T2, together with *z*-transformed age. The results are provided in Table 5. For the MMSE at T2, shown in the upper portion of Table 5, the lone significant predictor was the T2 TempOrd factor score (T2 PC TempOrd) with the significant regression equation, *F*(6, 90) = 3.1, *p* < .05, accounting for 11.7% of the variance (adjusted *r* ^2^). For the MoCA, shown in the middle of Table 5, a significant regression solution was obtained, *F*(6, 86) = 4.8, *p* < .01, accounting for 19.8% of the variance, with the T2 TempOrd factor score (T2 PC TempOrd) as the only significant predictor. For the final brief cognitive measure, the AQT, the regression equation was significant, *F*(6, 91) = 3.0, *p* < .05, and accounted for 11.2% of the variance (adjusted *r* ^2^) with low- and mid-frequency hearing loss in the right ear emerging as the only significant predictor. In summary, various combinations of auditory TempOrd processing and hearing loss at the time of cognitive assessment (T2) were significant predictors of performance on these brief measures of cognitive function. The variance explained by these auditory factors was relatively low, 10%–20%.

**Table 5.** Results of multiple linear-regression analyses with follow-up (T2) auditory principal component (PC) factor scores and *z*-transformed age as the independent variables and the dependent measures of follow-up (T2) *z*-transformed MMSE (top), MoCA (middle), and AQT (bottom) scores.

Dependent variable	Standardized	*t*	Sig.	Correlations		
	Beta			Zero-order	Partial	Part
**T2 MMSE**						
(Constant)		0.000	1.000			
T2Zage	−.152	−1.036	.303	−.229	−.109	−.099
T2PC HFHLbil	.025	0.202	.841	−.060	.021	.019
T2PC LMFHLrt	.012	0.113	.910	−.040	.012	.011
T2PC LMFHLlt	.099	0.997	.321	.072	.105	.096
T2PC TempOrd	−.338	−3.147	**.002**	−.389	−.315	−.302
T2PC GapDet	−.026	−0.269	.789	−.032	−.028	−.026
**T2 MoCA**						
(Constant)		0.000	1.000			
T2Zage	−.179	−1.254	.213	−.370	−.134	−.117
T2PC HFHLbil	−.091	−0.739	.462	−.191	−.079	−.069
T2PC LMFHLrt	−.006	−0.058	.954	−.068	−.006	−.005
T2PC LMFHLlt	−.091	−0.939	.350	−.123	−.101	−.088
T2PC TempOrd	−.360	−3.435	**.001**	−.419	−.347	−.321
T2PC GapDet	−.065	−0.696	.488	−.072	−.075	−.065
**T2 AQT**						
(Constant)		0.000	1.000			
T2Zage	.174	1.185	.239	.347	.123	.113
T2PC HFHLbil	.100	0.791	.431	.197	.083	.076
T2PC LMFHLrt	.222	2.053	**.043**	.282	.210	.196
T2PC LMFHLlt	−.004	−0.040	.968	.027	−.004	−.004
T2PC TempOrd	.123	1.145	.255	.181	.119	.110
T2PC GapDet	.040	0.422	.674	.047	.044	.040

*Note.* Significant t-test p values are shown in bold font. MMSE = Mini-Mental State Examination; HFHLbil = hearing loss at and above 2000 Hz bilaterally; LMFHLrt = hearing loss below 2000 Hz in the right ear; LMFHLlt = hearing loss below 2000 Hz in the left ear; MoCA = Montreal Cognitive Assessment; AQT = A Quick Test.

### Is Auditory Performance at Baseline (T1) or Rate of Decline in Auditory Function From T1 to T2 Predictive of the Rate of Decline in MMSE Scores Over the 9-Year Period?

This question could only be addressed for the MMSE as this was the only brief cognitive measure that was administered at *both* T1 and T2. The correlation between the MMSE scores at T1 and T2 was significant (*p* < .01) but relatively weak (*r* = .35). Due to this weak correlation, the linear slopes for the change in MMSE score from T1 to T2 are questionable, and this should be kept in mind when evaluating the associations between the rates of auditory and cognitive change over this 9-year period. When correlations were generated between the MMSE T1–T2 slope and corresponding slopes for age and all nine psychophysical auditory measures, all slopes expressed as *z* scores relative to the means and standard deviations for T1 (Salthouse, 2010b, 2011), none emerged as significant (*p* > .01). When linear-regression analysis was examined with the *z*-transformed T1–T2 slope for the MMSE as the dependent measure and T1 age and auditory measures as predictors, the regression equation was significant, *F*(6, 91) = 2.82, *p* < .05, and accounted for 10.1% of the variance (adjusted *r* ^2^), with the T1 TempOrd processing PC emerging as the only significant predictor (*p* < .01; partial and part correlations of *r* = −.30 and −.29, respectively). Thus, the only predictor of the rate of MMSE decline from T1 to T2 identified was TempOrd processing at T1.

## Summary and Conclusions

For the 98 older adults included in this longitudinal study, the MoCA emerges as the preferred cognitive screen for use by audiologists working with older adults. This statement is based on the somewhat stronger partial correlations in Table 2 showing associations of the MoCA with all three W3 PCs. That is, the breadth of cognitive assessment appears to be greater for the MoCA than the MMSE or the AQT. In addition, neither the MoCA nor the MMSE was associated with hearing loss measured at the same time (T2; see Table 5), at least for the severity of hearing loss among these 98 older adults, whereas this did impact the AQT score. In addition, there appeared to be finer gradations of cognitive function generated by the MoCA than the MMSE, with very few of the 98 participants in this study failing the MMSE (see Table 3). At this point, all that can be said regarding the use of the pass/fail data in Table 3 as a metric for comparison is simply that the tests differed. It is not possible to determine which was correct when they differed in pass/fail decisions as we have no other data to support a clinical diagnosis of cognitive impairment. Other studies, however, have found the MoCA to be more sensitive to the slight declines seen in mild cognitive impairment (Hoops et al., 2009; Nasreddine et al., 2005; Roalf et al., 2013). It should be noted that, effective September 1, 2020, a 1-hr online training and certification program will be required prior to use of the MoCA clinically (https://www.mocatest.org/mandatory-moca-test-training/). This should facilitate administration of the MoCA in a standardized fashion and make it more broadly accessible for use by audiologists working with older adults. Slavych (2019) recently provided a brief overview of the advantages and disadvantages of the MoCA and MMSE. The AQT is reserved primarily for assessment of concerns regarding cognitive PS, supported by the present analyses as well.

It is important to recognize that the effects documented in the prior literature on the association between hearing loss and cognition are quite small and only emerge as significant factors with very large study samples. This means that an audiologist should not expect reversal of cognitive decline with amplification for individual patients in the short term. Rather, this is a more important issue at a public health level where one tries to mitigate the number of risk factors for cognitive decline on a population basis, hearing loss being one of the most prominent (Livingston et al., 2017). Regardless, obtaining a brief, reliable assessment of cognitive function is critical when working with older adults with implications for the effective use and maintenance of hearing aids as well as interpreting hearing aid outcomes, such as aided speech understanding, for older adults.

All told, there were six linear-regression analyses performed to examine associations between either T1 or T2 auditory performance (and age) and T2 cognitive function measured with each of the three brief cognitive screens. All six regression solutions were significant, but no specific variables emerged as significant predictors in one of those analyses (T1 auditory with T2 AQT; see Table 4). Of the five remaining regression solutions, the auditory TempOrd processing PC emerged as the lone significant predictor and accounted for 10%–20% of the variance (adjusted *r* ^2^). For the only significant logistic regression analysis predicting pass/fail of the screen (MoCA), this same auditory TempOrd processing factor was the lone significant T2 beta coefficient. Recall that the TempOrd PC captures performance on the monaural two-item identification, dichotic two-item identification, and dichotic two-item location measures. It has long been known that dichotic listening includes both higher level auditory and nonmodality-specific cognitive factors (e.g., Bronkhorst, 2000, 2015; Cherry, 1953). Thus, it is perhaps not too surprising that this auditory factor, whether established 9 years prior (T1) or concurrently (T2), emerged most often as a predictor of cognitive function at T2. In fact, there has been renewed interest in this linkage since the pioneering work of Gates and colleagues (Gates et al., 2008, 2011, 2002, 1996) with recent meta-analyses and reviews available (e.g., Sardone et al., 2019; Yuan et al., 2018), as well as population studies of dichotic processing over the adult life span (Fischer et al., 2017). This linkage between measures of TempOrd identification and cognitive function was also observed in the original cross-sectional cohort of 245 young, middle-age, and older adults (Humes, Busey, et al., 2013). As noted then, and again here, this association is independent of both age and hearing loss (see Tables 4 and 5).

Somewhat surprising was the observation here that hearing loss itself seldom emerged as a significant explanatory factor for either subsequent or concurrent cognitive function. As noted in the introduction, there have been several cross-sectional and some longitudinal studies in aging that have observed an association. The only time hearing loss emerged as a significant factor here was in the T2 auditory and T2 AQT regression analyses (see Table 5). It is unclear why such associations were not observed more frequently in this study. Review of the correlations between the cognitive measure and each of the three hearing loss measures in the right-hand portions of Tables 4 and 5, however, shows that several approximated *r* = .1, which would be consistent with the grand average correlation between hearing loss and cognition from the systematic review by Loughrey et al. (2018). Typically, in the studies reviewed by Loughrey et al., hundreds of participants were included in each study and the pooled analyses represented results from thousands of older adults. With such large samples, *r* values of about .1 are statistically significant, whereas that was not the case in this smaller study of 98 older adults. The present linear-regression analyses with six predictors (age and five auditory factors) could detect significant regression solutions with an *r* ^2^ of .13 with 80% power (*p* = .05), and as noted above, most regression analyses were significant. However, the sample size may have been too small to detect significant partial effects of hearing loss on cognitive function, which, based on these analyses, were considerably smaller than the effects of TempOrd processing.

Finally, as noted in the Method section, older adults with hearing aids in this study wore their hearing aids during cognitive assessments and an assistive listening device was available for use by non–hearing aid users who had difficulty with the cognitive tests, all of which include auditory instructions and, except for the AQT, rely heavily on auditory stimuli for cognitive assessment. It is well known that hearing difficulties can impact performance on such auditory assessments by requiring greater cognitive resources to process the auditory instructions or stimuli, leaving fewer resources available for the actual cognitive task (e.g., Humes & Young, 2016; Pichora-Fuller et al., 2016). As noted above, there are versions of the MoCA available for those with severe hearing loss (V. Y. W. Lin et al., 2017), and a recent systematic review has been published on the effect of hearing loss on the MoCA (Utoomprurkporn et al., 2020). Studies such as these offer further support for the use of the MoCA by audiologists working with older adults, many of whom have impaired hearing.

## Supplementary Material

10.1044/2020_AJA-20-00077SMS1 Supplemental Material S1 A schematic of the stimulus sequences for all four TempOrd identification tasks, together with audio examples for the two monaural tasks.
