# Supplementary figures and images for: Associations Between Measures of Auditory Function and Brief Assessments of Cognition
Source: Am J Audiol. 2020 Sep 25;29(4):825–37. doi: 10.1044/2020_AJA-20-00077 (PMC8608158; doi:10.1044/2020_AJA-20-00077)

## Slide 1
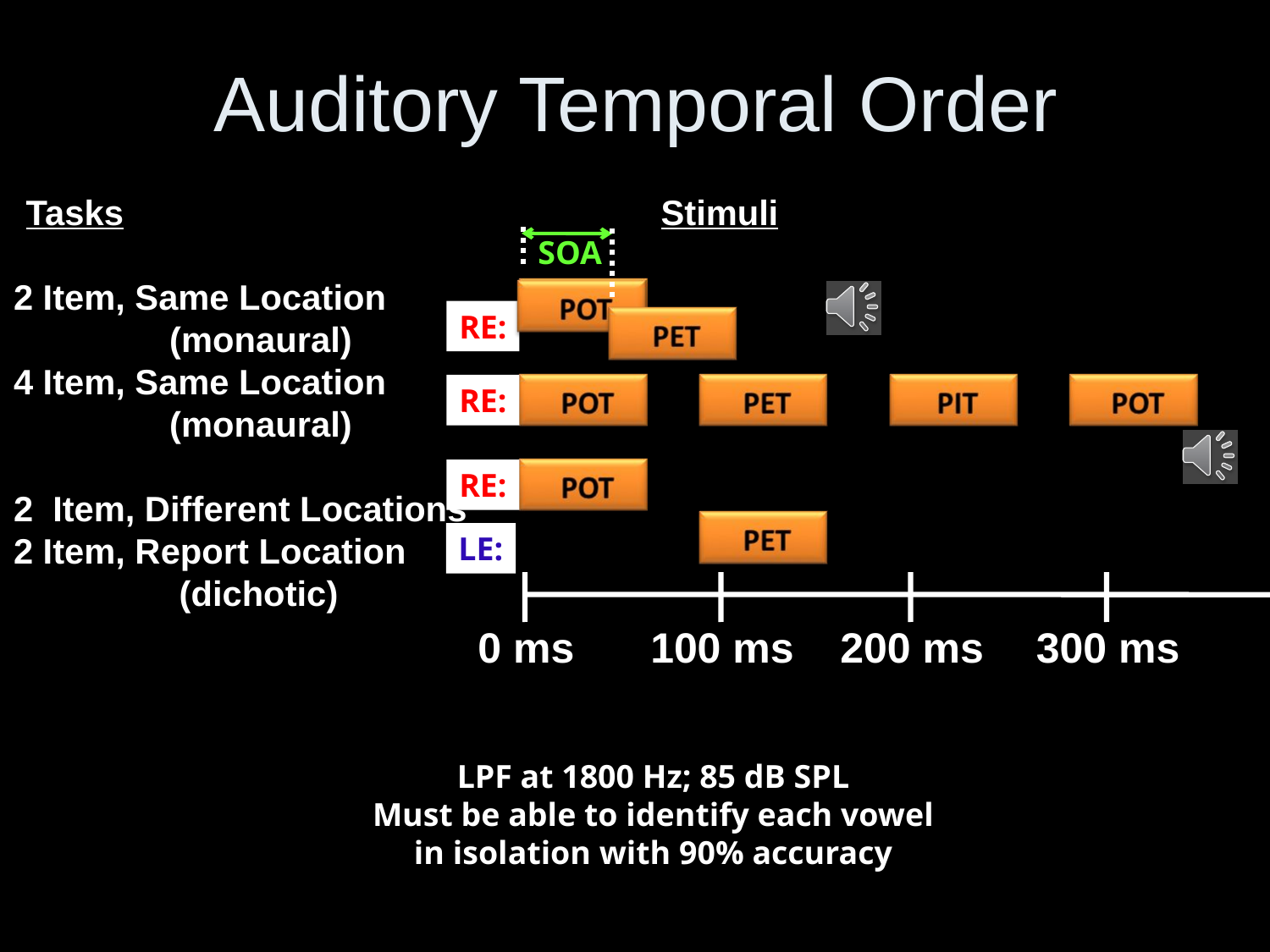

Supplement: Supplemental Material S1 [file AJA-29-825-s001.ppsx]
